# Patulin and LL‐Z1640‐2 induce apoptosis of cancer cells by decreasing endogenous protein levels of Zic family member 5

**DOI:** 10.1111/jcmm.17598

**Published:** 2022-10-25

**Authors:** Reiko Satow, Takeru Watanabe, Moeka Nomura, Shota Inagaki, Atsuko Yoneda, Kiyoko Fukami

**Affiliations:** ^1^ Laboratory of Genome and Biosignals Tokyo University of Pharmacy and Life Sciences Hachioji, Tokyo Japan

**Keywords:** apoptosis, LL‐Z1640‐2, melanoma, pancreatic cancer, patulin, ZIC5

## Abstract

Zic family member 5 (ZIC5) is a transcription factor that promotes the survival of several cancer cell types. As ZIC5 is expressed at minimal levels in normal human adult tissues, it is a potential therapeutic target. In this study, we screened a chemical library containing 3398 compounds that includes pre‐existing drugs and compounds with known effects to identify ZIC5 inhibitors. In the first screening, 18 hit compounds decreased GFP intensity in melanoma A375 cells overexpressing GFP‐tagged ZIC5. In the second screening, five compounds that attenuated ZIC5 protein levels in A375 cells were identified. Among them, LL‐Z1640‐2 and patulin selectively induced apoptosis in melanoma cells expressing ZIC5, while only inducing very low levels of apoptosis in normal human melanocytes, which have no detectable *ZIC5* expression. LL‐Z1640‐2 and patulin also induced apoptosis in BRAF inhibitor‐resistant melanoma, pancreatic cancer, cholangiocarcinoma and colorectal cancer cells. LL‐Z1640‐2‐ and patulin‐mediated suppression of melanoma proliferation were rescued by ZIC5 overexpression. These results suggest that LL‐Z1640‐2 and patulin are promising compounds that decrease ZIC5 expression to induce apoptosis in cancer cells.

## 1 INTRODUCTION

Despite recent advances in molecular targeted therapies and immunotherapies, metastatic melanoma is still a highly aggressive disease with a poor prognosis. Because approximately 50% of melanomas harbour an activating mutation in the *BRAF* gene, several BRAF inhibitors have been developed and adopted as therapeutic agents that are effective against melanoma with *BRAF* mutations. Recently, the BRAF‐targeted approach has shifted to combination therapy with MEK inhibitors and/or immunotherapy, which has dramatically improved patient outcomes.^1^, ^2^ However, the effectiveness of these approaches is limited in some patients because of primary or acquired resistance to treatment.^3^ Resistance to BRAF‐targeted treatment frequently occurs because residual tumour cells acquire resistance to BRAF inhibitors by reactivating the MAPK pathway.^3^ Thus, the identification of novel drugs that can induce melanoma cell death at a high rate and/or are effective against BRAF inhibitor‐resistant melanoma is required.

Previously, we screened neural crest‐associated genes and identified the Zic family member 5 (ZIC5) as a critical transcription factor for melanoma survival and drug resistance.^4^ ZIC5 expression is enhanced in melanoma tissues, while *ZIC5* expression is barely detectable in most human adult tissues, except in the testes and cerebral cortex (The Human Protein Atlas). ZIC5 activates signal transducer and activator of transcription 3 (STAT3), which is known to be associated with drug resistance in many types of cancers, by promoting anti‐apoptotic factors such as BCL‐XL.^5^ ZIC5 knockdown induced apoptosis in melanoma cells and synergistically enhanced apoptosis when using a BRAF inhibitor.^4^ ZIC5 knockdown also induced cell death in BRAF inhibitor‐resistant melanoma cells.^4^

*ZIC5* expression is also enhanced in colorectal cancer and is associated with the tumour pathological stage. ZIC5 positively regulates proliferation, survival and primary drug resistance in these cells.^6^ Recently, we disclosed that the overall survival of patients with ZIC5‐expressing pancreatic cancer was significantly lower than that of patients without ZIC5 expression.^7^ ZIC5 knockdown induces apoptosis in colorectal cancer, pancreatic cancer and cholangiocarcinoma cells and additively or synergistically induces apoptosis with anti‐cancer agents such as oxaliplatin and gemcitabine.^6^, ^7^ ZIC5 knockdown also inhibits proliferation and tumour growth in non‐small cell lung cancer and hepatocellular carcinoma.^8^, ^9^ *ZIC5* has been shown to be a target of miR‐761, which is suppressed by circRNA hsa_circ_0007534 in gliomas. The circRNA hsa_circ_0007534 is highly expressed in glioma tissues compared with normal brain tissues and promotes the proliferation and migration of glioma cells.^10^

Thus, ZIC5 is associated with the survival and malignant phenotypes of cancer cells. However, a small compound that inhibits or decreases ZIC5 expression has yet to be identified. In this study, we screened a validated chemical library containing 3398 pre‐existing compounds to identify molecules that decreased levels of ZIC5.

## 2 MATERIALS AND METHODS

### 2.1 Cells and cultures

Cell lines used in this study were obtained and maintained as previously described.^4^, ^6^

### 2.2 Plasmid construction and transfection

Sequences encoding GFP and ZIC5 were amplified and cloned into the pEBMulti‐Puro vector (FUJIFILM Wako Pure Chemical Corporation) (GFP‐ZIC5). In the first screening, the constructed plasmid was transfected into A375 cells using polyethylenimine (PEI) (Polysciences). In the second screening, the ZIC5/pcDNA described previously^4^ was transfected into A375 cells using PEI. To obtain stable cells for the rescue experiments, transfected cells were selected with 1 mg/ml G418 (10131035, Thermo Fisher Scientific) for 12 days, and the resulting bulk cells were analysed.

### 2.3 Small compound screening

In the first screening, A375 cells overexpressing GFP‐ZIC5 were plated in 96‐well plates at a density of 500 cells/well. The library compounds were added at a concentration of 10 μM in duplicate. After 15–20 h, GFP intensity was assessed using an In Cell Analyser 2000 (GE Healthcare) with fluorescein isothiocyanate (FITC) filters. GFP intensity in each well was determined using the In Cell Analyser Workstation 3.7 software (GE Healthcare). In the second screening, A375 cells overexpressing ZIC5 were plated in 96‐well plates at a density of 500 cells/well. The library compounds were added at concentrations ranging from 0.1 to 10 μM. After 15–20 h, cells were fixed with methanol for 10 min at −20°C, blocked with 5% bovine serum albumin (BSA) in PBS with 0.05% Tween‐20 (TPBS) and incubated with anti‐ZIC5 antibody (GTX104840; GeneTex) overnight at 4°C. Anti‐rabbit IgG‐conjugated Alexa Fluor 594 (Thermo Fisher Scientific) was used as a secondary antibody. The ZIC5 intensity was assessed using an In Cell Analyser 2000 (GE Healthcare). ZIC5 intensity in each well was determined using the In Cell Analyser Workstation 3.7 software (GE Healthcare).

### 2.4 Apoptosis assays

The cells were incubated with CellEvent Caspase‐3/7 Green Detection Reagent (Invitrogen) and Hoechst33342 (Dojindo) for 30 min. Cells were analysed using the In Cell Analyser 2000 with 4′,6‐diamidino‐2‐phenylindole (DAPI) and FITC filters. The ratio of caspase‐3/7‐positive cells to Hoechst33342‐positive cells was determined using the In Cell Analyser Workstation 3.7 software (GE Healthcare).

### 2.5 Cell proliferation assays

Cell proliferation assays were performed as described previously.^4^

### 2.6 Reagents

Patulin (Santa Cruz Biotechnology), LL‐Z1640‐2 (Santa Cruz Biotechnology) and PLX4032 (Selleck Chemicals) were obtained from commercial sources as denoted and used at the indicated concentrations.

### 2.7 Western blot analysis

Western blotting was performed as described previously.^11^ Primary antibodies against ZIC5 (ARP33669; Aviva Systems Biology), GAPDH and phospho‐STAT3 (Tyr705) (Catalogue# 9145; Cell Signalling Technology) were used. GAPDH was used as a loading control. Images were obtained using LuminoGraph I (ATTO). The signal intensity was quantified using a CS analyser (ATTO).

### 2.8 Affinity check

To evaluate the binding between ZIC5 protein and LL‐Z1640‐2 or patulin, we evaluated temperature‐related intensity change (TRIC), which is derived from microscale thermophoresis (MST) using Dianthus NT.23Pico^12^ (NanoTemper Technologies GmbH). Purified HIS‐ZIC5 was labelled using the Monolith His‐Tag Labeling Kit RED‐tris‐NTA 2nd Generation (NanoTemper Technologies). The labelled HIS‐ZIC5 was diluted with PBS and mixed in the total volume of 20 μl per well with or without 100 μM LL‐Z1640‐2 or patulin. The prepared 384‐well plates (NanoTemper Technologies) were placed in the instrument, and data acquisition was done at 25°C along with a sequence of 5 s of laser irradiation. Acquired data were analysed using the DI.Screening Analysis software (NanoTemper Technologies), to calculate the area response values, which represent the TRIC difference between the samples.^12^

### 2.9 Statistical analysis

Statistical analyses were performed using the R statistical software package (v. 4.0.3). Data are presented as the mean ± standard deviation (SD) in the bar graphs, unless otherwise indicated. The significance of differences was determined using the statistical tests indicated in the individual figure legends. Statistical significance was set at *p* < 0.05.

## 3 RESULTS

### 3.1 Several candidate compounds that reduce ZIC5 expression levels were identified

**FIGURE 1:**
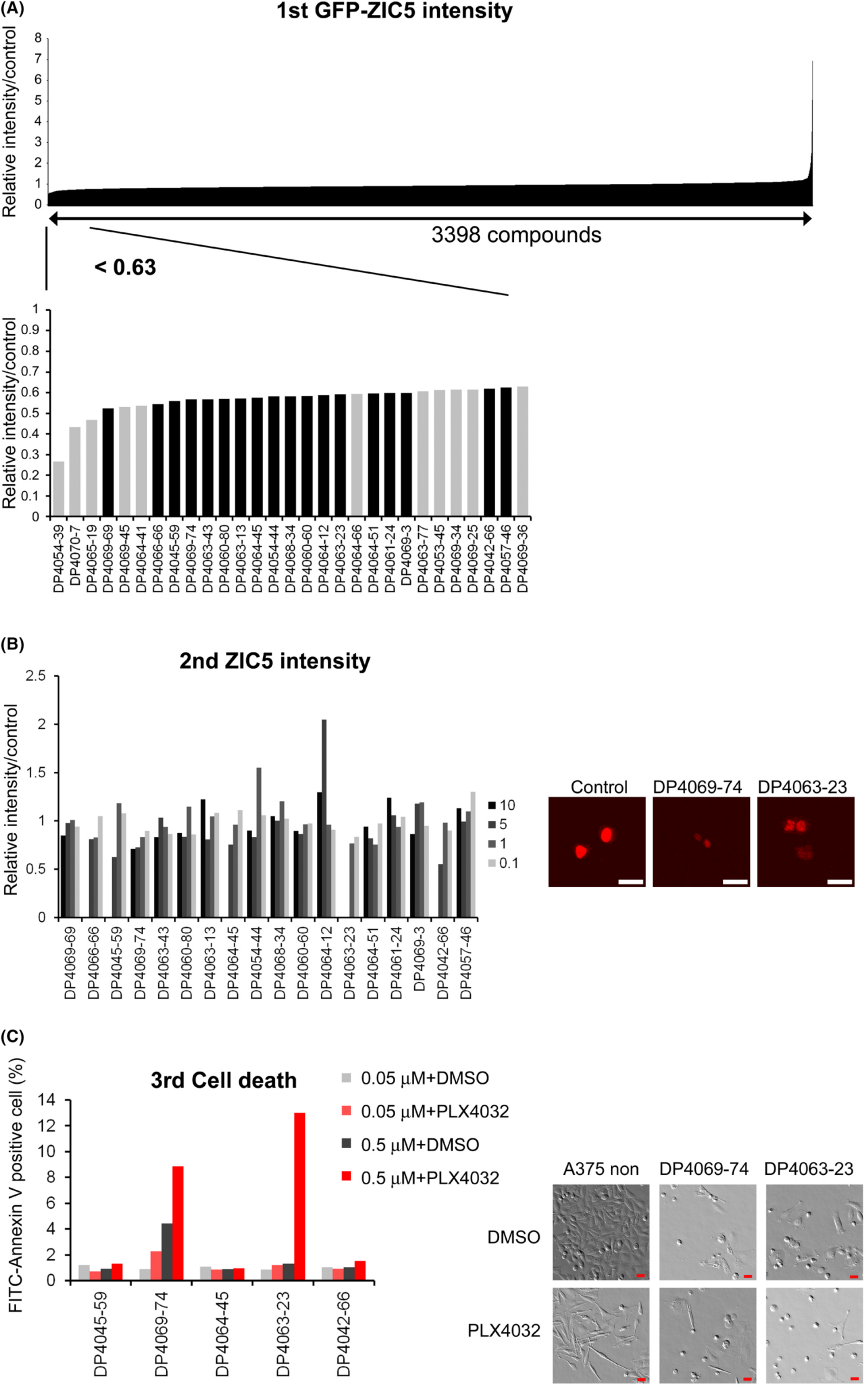
Identification of several candidate compounds that reduce ZIC5 protein levels. (A) In the first screening, A375 melanoma cells overexpressing GFP‐ZIC5 were treated with 3398 compounds from the validated compound library at a concentration of 10 μM for 15–20 h in duplicate. The average GFP intensity is shown. The black bar in the graph below shows that both wells meet the criteria (<Av‐2SD [0.63] and are not produced by a non‐specific signal). (B) In the second screening, A375 melanoma cells overexpressing ZIC5 were treated with the first 18 hit compounds at concentrations of 0.1, 1, 5 or 10 μM for 15–20 h in duplicate. The ZIC5 intensity was quantified, and the average is shown. Highly toxic concentrations were removed. Obtained images of DP4069‐74 (LL‐Z1640‐2) and DP4063‐23 (patulin) are shown. Scale bar indicates 30 μm. (C) In the third screening, A375 melanoma cells were pre‐treated with the second hit compounds at 0.05 or 0.5 μM for 24 h followed by treatment with PLX4032 (0.5 μM) for 48 h. The percentage of FITC‐annexin V positive cells is shown. Obtained phase‐contrast images of cells treated with DP4069‐74 (LL‐Z1640‐2) and DP4063‐23 (patulin) (0.5 μM) are also shown. Scale bar indicates 30 μm.

To identify compounds that reduce the levels of ZIC5 protein, we used a validated chemical compound library that includes pre‐existing drugs (Open Innovation Center for Drug Discovery, The University of Tokyo, Japan). We found that 29 of 3398 compounds reduced GFP intensity to 63% (Av‐2SD) that of the control or less in A375 melanoma cells overexpressing GFP‐tagged ZIC5 (Figure 1A). Among them, 18 compounds reproducibly reduced GFP intensity and were confirmed not to have resulted from a non‐specific signal (by assessing the fluorescent images that were taken during screening) (Figure 1A, black bar). In the second screen, five compounds reproducibly reduced the level of ZIC5 (not tagged) that was overexpressed in A375 cells (Figure 1B). Because ZIC5 knockdown induced melanoma cell death synergistically with the BRAF inhibitor,^4^ A375 melanoma cells were treated with the five candidate compounds with or without the BRAF inhibitor (PLX4032) to assess apoptosis. Two of the five identified compounds induced cell death synergistically with PLX4032 (Figure 1C); thus, these screens identified two candidate compounds (patulin and LL‐Z1640‐2) that reduce ZIC5 protein expressions levels and induce melanoma cell death.

### 3.2 LL‐Z1640‐2 and patulin reduce ZIC5 levels in A375 melanoma cells in a dose‐dependent manner

**FIGURE 2:**
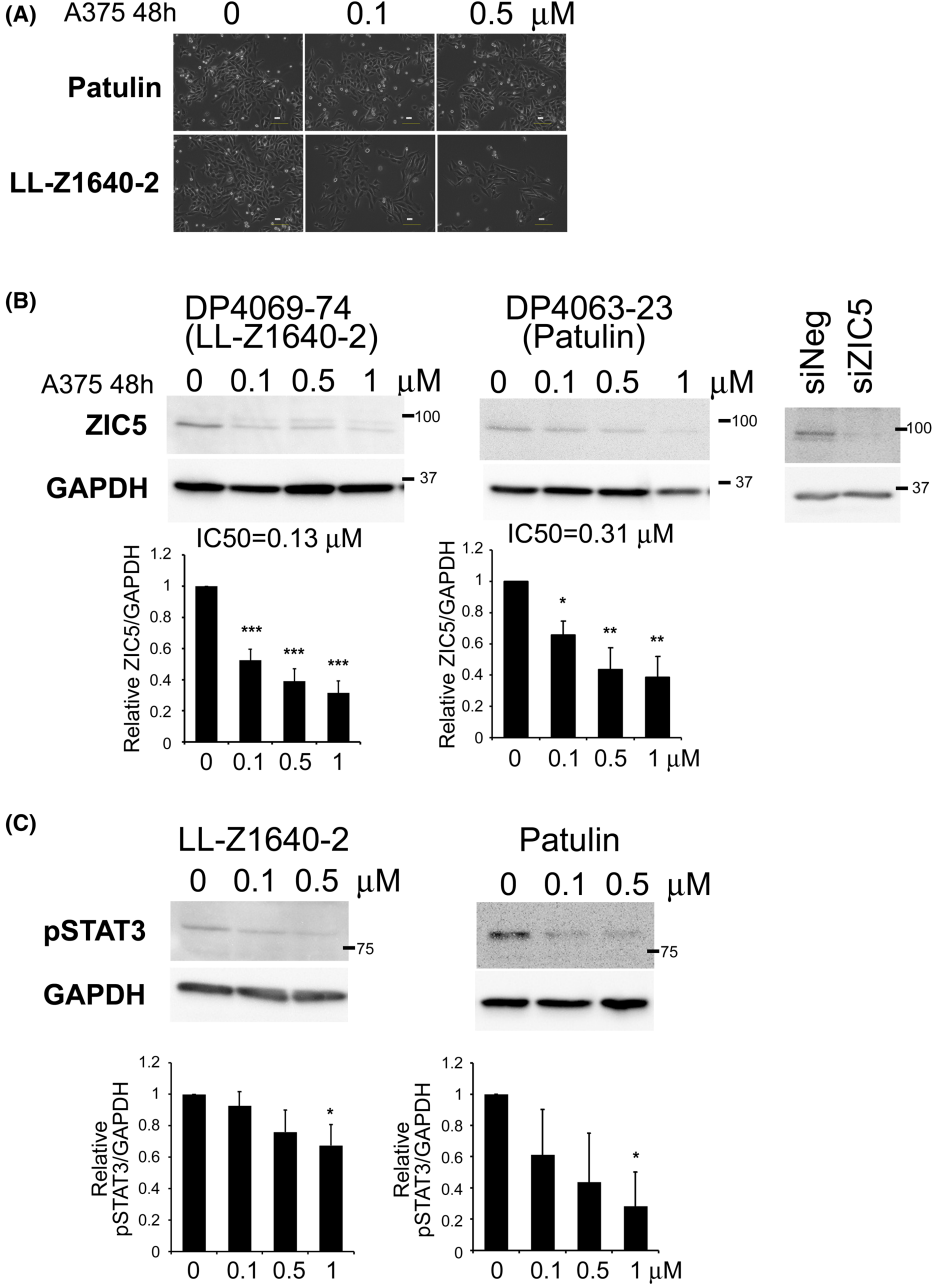
LL‐Z1640‐2 and patulin reduce ZIC5 protein levels in A375 melanoma cells in a dose‐dependent manner. (A) A375 cells were treated with LL‐Z1640‐2 and patulin at 0.1 or 0.5 μM for 48 h. Phase‐contrast images of cells are shown. (B) Western blotting of ZIC5 and GAPDH (as an internal control) is shown. Band intensities were quantified and normalized to GAPDH levels. The results shown are the mean ± SD of three independent experiments. Statistical analysis was performed using a two‐tailed Dunnett's multiple comparison of means test (***p < 0.001, **p < 0.01, *p < 0.05). Positive control staining of ZIC5 in A375 cells transfected with negative control siRNA (siNeg) or siRNA targeting ZIC5 (siZIC5) is shown in right panels. (C) Western blotting of phosphorylated STAT3 (Tyr705) and GAPDH (as an internal control) is shown. Representative images of two independent experiments are shown.

To investigate whether LL‐Z1640‐2 and patulin reduce endogenous ZIC5 protein levels, A375 cells were treated with these compounds at concentrations ranging from 0.1 to 1 μM for 48 h. Western blotting revealed that LL‐Z1640‐2 and patulin reduced endogenous ZIC5 levels in a dose‐dependent manner (Figure 2A,B). Moreover, phosphorylation of STAT3, which is activated downstream of ZIC5 and promotes drug resistance, was reduced by treatment with either LL‐Z1640‐2 or patulin (Figure 2C).

### 3.3 LL‐Z1640‐2 and patulin induce apoptosis in BRAF inhibitor‐resistant melanoma cells and ZIC5‐expressing melanoma cells

**FIGURE 3:**
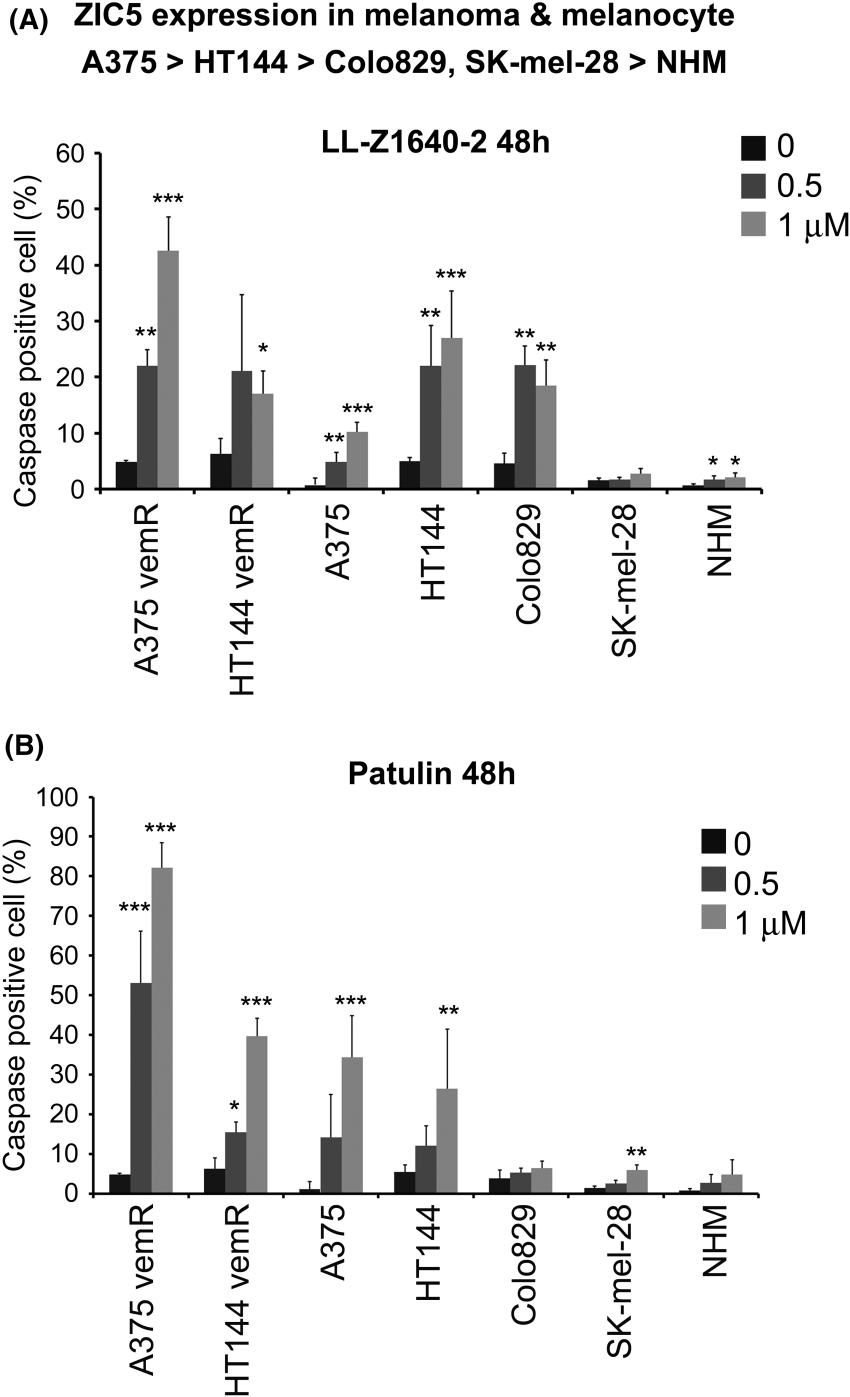
LL‐Z1640‐2 and patulin induce apoptosis in BRAF inhibitor‐resistant melanoma cells and melanoma cells expressing ZIC5. (A, B) A375, HT144, *Colo*‐829, SK‐Mel‐28, NHM and BRAF inhibitor‐resistant cell lines A375 vemR and HT144 vemR were treated with LL‐Z1640‐2 or patulin at the indicated dose for 48 h. Apoptosis assays revealed the percentage of caspase‐3/7 positive cells from three independent experiments. Statistical analysis was performed using the two‐tailed Dunnett's multiple comparison test (***p < 0.001, **p < 0.01, *p < 0.05).

We assessed the effect of LL‐Z1640‐2 and patulin in the melanoma cell lines A375, HT144, COLO829 and SK‐MEL‐28, the BRAF inhibitor‐resistant cell lines established in our previous study (A375vemR‐3 and HT144vemR‐3),^4^ and normal human melanocytes (NHMs). Among them, A375 and HT144 (and their derivatives A375 and HT144 vemR) expressed high levels of ZIC5,^4^ whereas NHMs showed no detectable *ZIC5* expression.^4^ A375vemR‐3 and HT144vemR‐3 shows higher ZIC5 expression than parental cells.^4^ Treatment with LL‐Z1640‐2 for 48 h induced apoptosis in 10%–30% of A375, HT144 and COLO829 cells at 1 μM, whereas apoptosis was induced in <3% of NHM and SK‐MEL‐28 cells by LL‐Z1640‐2 (Figure 3A). Treatment with patulin for 48 h induced apoptosis in 30%–40% of A375 and HT144 cells at 1 μM, whereas apoptosis was induced in <10% of NHM, SK‐MEL‐28 and COLO829 cells by patulin (Figure 3B). Furthermore, LL‐Z1640‐2 induced apoptosis in 40% and 20% of A375 vemR and HT144 vemR cells, respectively (Figure 3A). Patulin induced apoptosis in 80% and 40% of A375 vemR and HT144 vemR cells, respectively (Figure 3A). These results suggest that LL‐Z1640‐2 and patulin induce apoptosis in BRAF inhibitor‐resistant melanoma cells and that melanoma cells with high ZIC5 expression levels tend to be sensitive to these compounds.

### 3.4 LL‐Z1640‐2 and patulin reduce melanoma growth synergistically with a BRAF inhibitor

**FIGURE 4:**
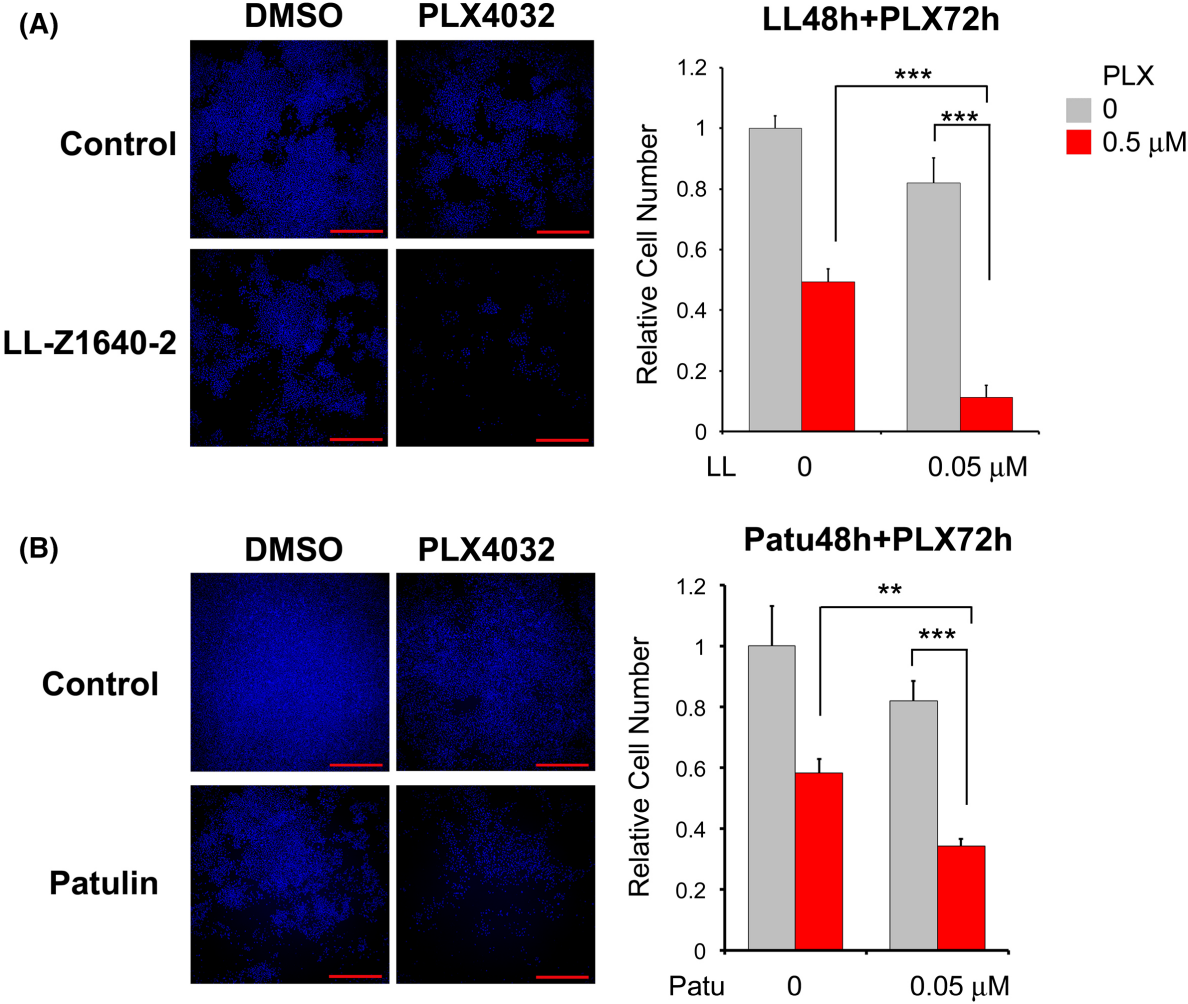
LL‐Z1640‐2 and patulin reduce melanoma growth synergistically with a BRAF inhibitor. (A, B) A375 was pre‐treated with LL‐Z1640‐2 or patulin for 48 h followed by PLX4032 for 72 h. Cells were stained with Hoechst and the cell number was determined by an IN Cell Analyser 2000. Obtained images are shown (Bar indicates 1000 μm). The relative cell number is shown in the bar graphs. Statistical analysis was performed using the two‐tailed Tukey's multiple comparison test (*n* = 4; ***p < 0.001, **p < 0.01).

ZIC5 knockdown induced melanoma cell death synergistically with the BRAF inhibitor PLX4032^4^; accordingly, we assessed if a synergistic effect would be produced by treatment with low doses of LL‐Z1640‐2 or patulin with PLX4032. When A375 cells were pretreated with LL‐Z1640‐2 or patulin for 48 h, followed by PLX4032 for 72 h, the cell number was significantly reduced compared with that in cells treated with single compounds (Figure 4A,B).

### 3.5 Overexpression of ZIC5 rescues the LL‐Z1640‐2‐ or patulin‐mediated reduction of melanoma proliferation

**FIGURE 5:**
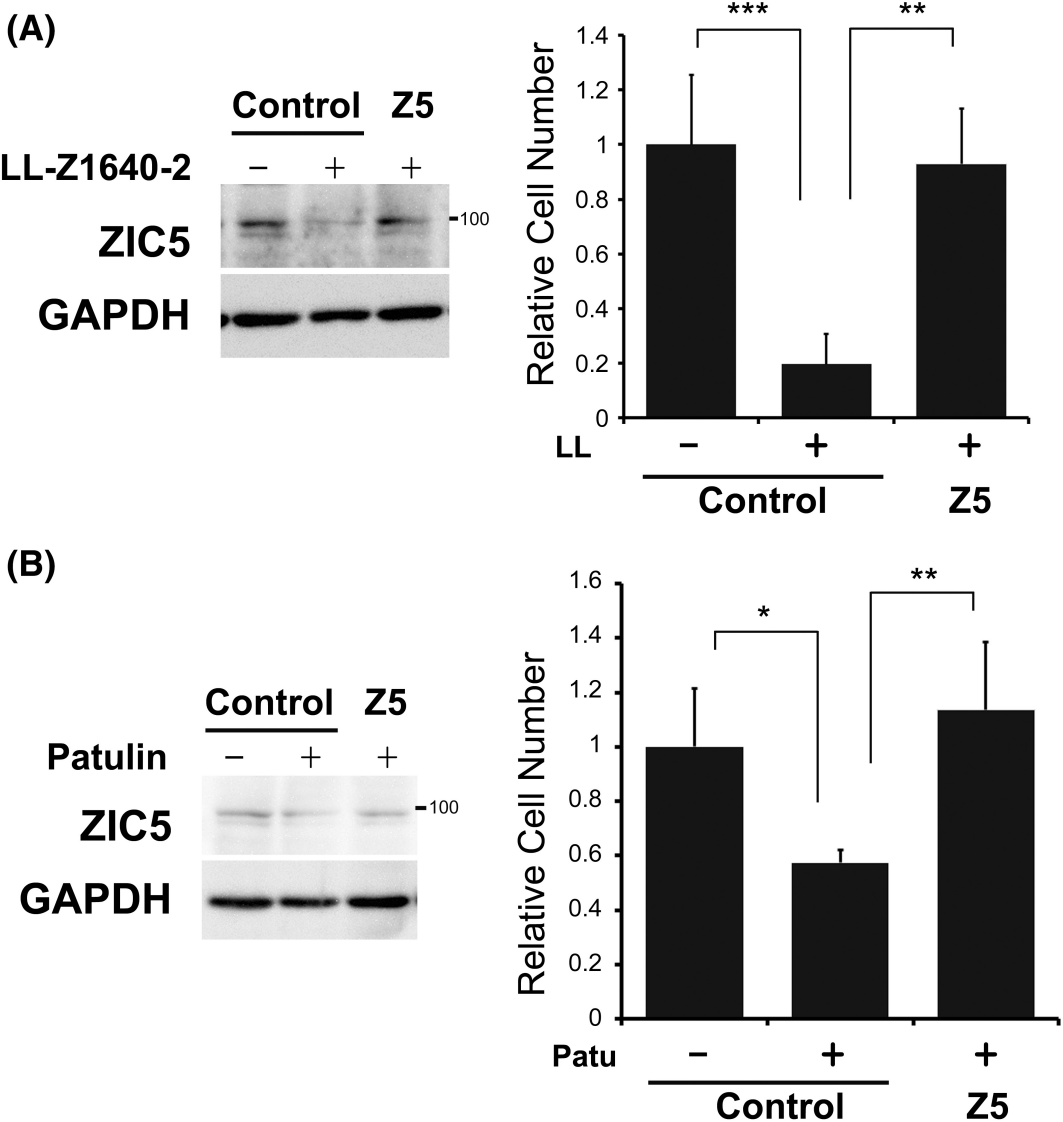
Overexpression of ZIC5 rescues the LL‐Z1640‐2‐ or patulin‐mediated reduction of melanoma proliferation. A375 cells transfected with a control vector (control) or a *ZIC5*‐expression vector (Z5) were selected and plated. The cells were treated with LL‐Z1640‐2 (A) or patulin (B), as indicated. After 2 days, the cells were treated with PLX4032 for 72 h. Cells were stained with Hoechst and the number of cells was determined. (Left) Western blotting of ZIC5 and GAPDH (internal control). (Right) The relative number of cells are shown. Statistical analysis was performed using a two‐tailed Tukey's multiple comparison test (*n* = 4; *** *p* < 0.001, ** *p* < 0.01, *p < 0.05).

To elucidate whether LL‐Z1640‐2 and patulin induced apoptosis via a reduction in the protein levels of ZIC5, we overexpressed ZIC5 to rescue the ZIC5 protein level. When ZIC5 was overexpressed in the presence of LL‐Z1640‐2 or patulin, restoring the ZIC5 protein level to that of endogenous levels, the reduction in cell number observed in A375 cells treated with LL‐Z1640‐2 or patulin was negated (Figure 5A,B). These results suggest that patulin and LL‐Z1640‐2 reduced the number of ZIC5‐expressing melanoma cells by reducing ZIC5 protein levels.

### 3.6 Patulin and LL‐Z1640‐2 induce apoptosis in pancreatic cancer, cholangiocarcinoma and colorectal cancer cells

**FIGURE 6:**
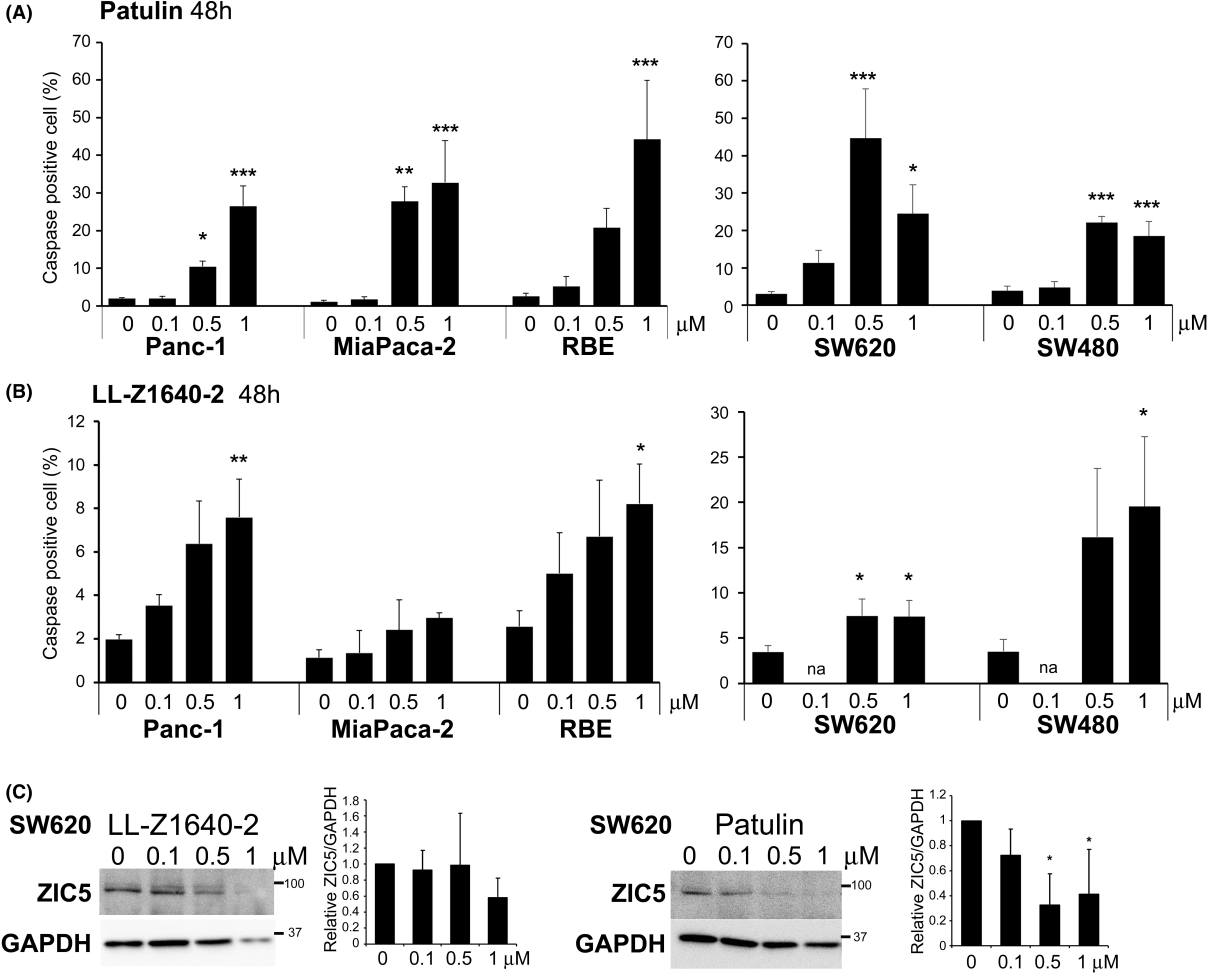
LL‐Z1640‐2 and patulin induce apoptosis in pancreatic carcinoma, cholangiocarcinoma and colorectal cancer cells. Pancreatic carcinoma cell lines Panc‐1 and MiaPaca‐2, cholangiocarcinoma cell line RBE and colorectal cancer cell lines SW620 and SW480 were treated with patulin (A) or LL‐Z1640‐2 (B) for 48 h, and the percentage of caspase‐3/7 positive cells from three independent apoptosis assays is shown. (C) Western blotting of ZIC5 and GAPDH (as an internal control) is shown. Band intensities were quantified and normalized to GAPDH levels. The results shown are the mean ± SD of three independent experiments. Statistical analysis was performed using a two‐tailed Dunnett's multiple comparison test (***p < 0.001, **p < 0.01, *p < 0.05).

Because ZIC5 knockdown induces apoptosis in pancreatic cancer, cholangiocarcinoma and colorectal cancer cells,^7^ we assessed the effect of patulin and LL‐Z1640‐2 on these cell lines. Patulin and/or LL‐Z1640‐2 indeed induced apoptosis in pancreatic cancer cells (Panc‐1 or MiaPaca‐2), cholangiocarcinoma cells (RBE) and colorectal cancer cells (SW620 and SW480) (Figure 6A,B). Western blot analysis revealed that patulin significantly reduced the ZIC5 protein at 0.5 and 1 μM in SW620 cells, while LL‐Z1640‐2 could not reduce the ZIC5 protein significantly (Figure 6C). These results indicate that patulin and LL‐Z1640‐2 are potential therapeutic compounds for many types of cancers.

### 3.7 Patulin and LL‐Z1640‐2 show no direct binding with ZIC5 protein

**FIGURE 7:**
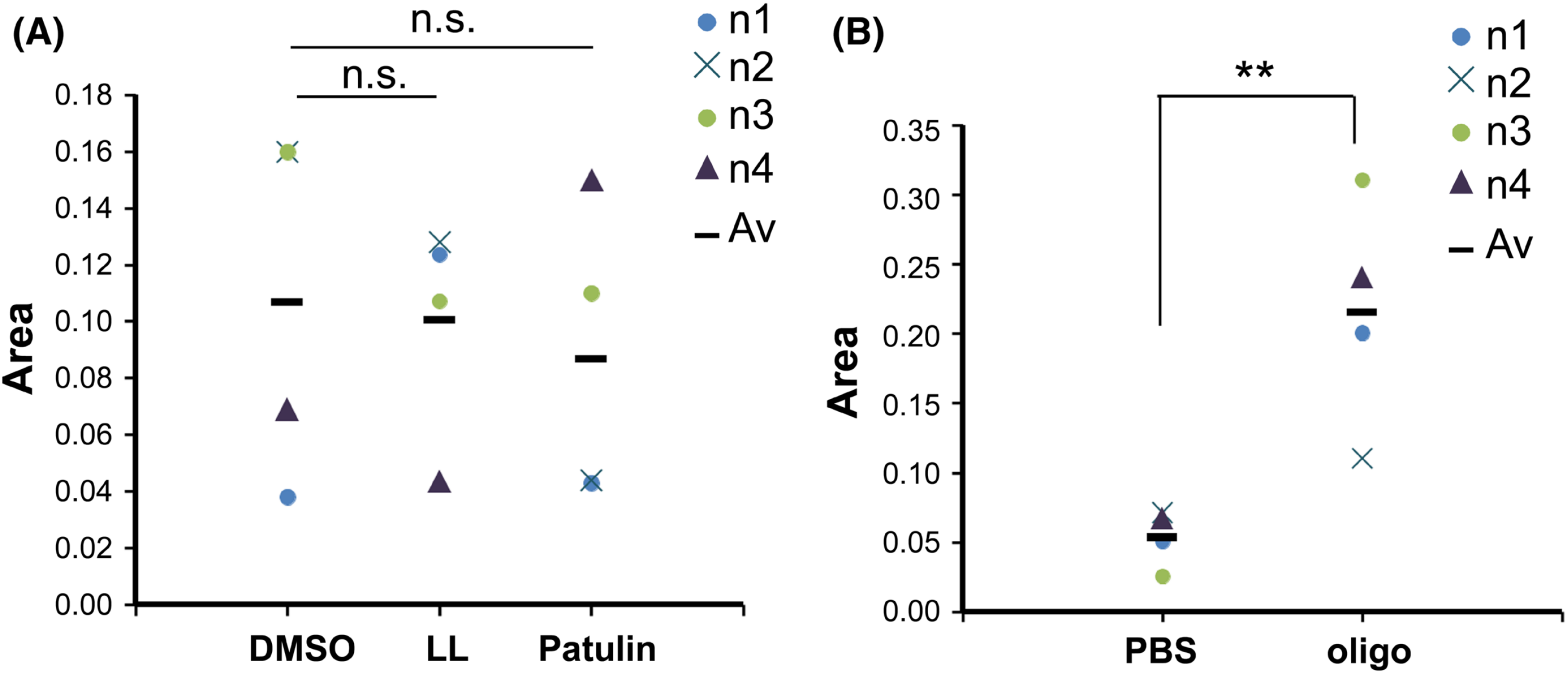
LL‐Z1640‐2 and patulin show no direct interaction with ZIC5 protein. (A) The labelled HIS‐ZIC5 was diluted in PBS with or without 100 μM LL‐Z1640‐2 or patulin. The TRIC was obtained using Dianthus and analysed using the DI.Screening Analysis software (NanoTemper Technologies). Area (area response values) represents the TRIC difference between the samples. Area of DMSO represents the TRIC difference between the solvent only control samples (mixed with HIS‐ZIC5), while the area of LL‐Z1640‐2 or patulin is the difference between the solvent only control sample (mixed with HIS‐ZIC5) and the compound sample (mixed with HIS‐ZIC5). (B) Positive control experiments were performed using ZIC5‐binding oligonucleotide (TGTGATTTTCGTCTTGGGTGGTCTCCCTCG).^24^ Statistical analysis was performed using a two‐tailed Dunnett's multiple comparison test (**p < 0.01).

To clarify whether Patulin and LL‐Z1640‐2 bind to ZIC5, we assessed their affinity using an MST based method.^12^ These compounds did not affect the TRIC signal of HIS‐ZIC5, suggesting that these compounds are not directly bound to ZIC5 (Figure 7).

## 4 DISCUSSION

In this study, we explored compounds with known effects to identify potential inhibitors of ZIC5. We found that patulin and LL‐Z1640‐2 reduced ZIC5 protein levels and induced apoptosis in many tumour cell lines, including BRAF inhibitor‐resistant melanoma cells, but not in NHMs. Both LL‐Z1640‐2 and patulin reduced ZIC5 protein, which is overexpressed by general promoter systems (CAG promoter [Figure 1A] or CMV promoter [Figure 1B]); thus, both compounds may reduce ZIC5 protein expression after transcription.

Patulin (4‐hydroxy‐4H‐furo [3,2‐c] pyran‐2(6H)‐one) is a mycotoxin primarily produced by species of the *Penicillium*, *Aspergillus* and *Byssochlamys* genera. Patulin is found in fruits such as apples, pears and grapes.^13^, ^14^ This food toxin can be a serious health concern, but the International Agency for Research on Cancer has categorized patulin in Group 3, meaning that it is considered non‐carcinogenic. The European Commission and FAO/WHO Joint Expert Committee on Food Additives and Contaminants have established the maximum permitted limits and suggested a provisional maximum tolerable daily intake of 0.4 mg/kg body weight/day for patulin.^13^, ^14^ The tumour regression effect of patulin in melanoma‐bearing mice was reported to be within this range. Administration of 5 μg/kg of patulin every 2 days to mice significantly suppressed melanoma growth by inducing ER stress and apoptosis.^15^ It has also been reported that patulin exerts its toxic effect by covalently binding to reactive sulfhydryl groups in cellular proteins, as well as by glutathione depletion, resulting in oxidative damage and the generation of reactive oxygen species (ROS).^16^, ^17^ Therefore, the effect of patulin and its partial mechanism of action has been reported with regards to melanoma growth in vivo. Our results provide new insights into the effects of patulin and suggest that patulin is a promising anti‐cancer reagent for many types of tumour cells that express ZIC5.

LL‐Z1640‐2 (also known as 5Z‐7‐oxozeaenol) is a resorcylic acid lactone that is produced by various fungal strains.^18^ LL‐Z1640‐2 is an inhibitor of transforming growth factor‐activated kinase 1 (TAK1).^18^ LL‐Z1640‐2 has been shown to block interleukin‐1‐induced activation of TAK1 and activation of TAK1‐downstream proteins, such as JNK/p38 MAPK and NF‐κB.^18^ LL‐Z1640‐2 has been shown to attenuate CRC cell viability, cell growth rate and cell growth in vivo.^19^ Because the growth inhibition by LL‐Z1640‐2 is reversed by adding a non‐specific thiol antioxidant, LL‐Z1640‐2‐induced oxidative stress is important for the inhibition of colorectal cancer cell growth.^19^ However, TAK1 inhibitors other than LL‐Z1640‐2 did not suppress ZIC5 expression (data not shown), suggesting that LL‐Z1640‐2 may suppress ZIC5 via TAK1‐independent mechanisms. Our results provide new insights into the effects of LL‐Z1640‐2 and suggest that this compound is a promising anti‐cancer reagent for many types of tumour cells that express ZIC5.

As mentioned above, both patulin and LL‐Z1640‐2 are associated with ROS generation and ROS often affect protein function and/or stability.^20^, ^21^ ROS have been reported to have dual roles in promoting cell survival and cell death.^22^ ROS can also induce apoptosis in cancer cells.^22^ Although the mechanism by which patulin or LL‐Z1640‐2 reduces ZIC5 protein levels was not elucidated in our study, this might be attributed to ROS production. As seen in Figure 2B, the treatment of A375 with 1 μM LL‐Z1640‐2 or patulin reduced ZIC5 protein levels to 0.32 or 0.39 (relative to 0 μM control), respectively. However, treatment with 1 μM patulin induced apoptosis (34.4%) to a greater extent than that with LL‐Z1640‐2 (10.2%) (Figure 3). Moreover, quantitative analysis of phospho‐STAT3, which is an important anti‐apoptotic factor activated by ZIC5, revealed that patulin reduced phospho‐STAT3 more efficiently than LL‐Z1640‐2 (Figure 2C). These results suggest that factors affected by patulin, other than ZIC5, might also be involved in the induction of apoptosis, or factors influenced by LL, other than ZIC5, might inhibit the induction of apoptosis.

In SW620 cells, 0.5 μM patulin induced apoptosis in 45% of population, while 0.5 μM LL‐Z1640‐2 induced apoptosis only in 7.5% of population (Figure 6A,B). Western blot analysis revealed that patulin significantly reduced the ZIC5 protein levels at 0.5 μM, while LL‐Z1640‐2 could not reduce the ZIC5 protein levels at 0.5 μM (Figure 6C), showing consistency in the apoptosis inducing effect. However, because LL‐Z1640‐2 slightly induced apoptosis in SW620 cells, it is speculated that the apoptosis‐inducing effect of LL‐Z1640‐2 on SW620 is not mediated by ZIC5 reduction, but by other molecular pathways. These results suggest that apoptosis is highly induced when ZIC5 protein is reduced by compounds; however, other compound‐affected factors are also involved in cancer cell survival.

The poor solubility of LL‐Z1640‐2 limits its in vivo bioavailability; this could be solved through nanoparticle‐mediated delivery.^23^ Furthermore, we have conducted an in vivo experiment using patulin with nude mice bearing human melanoma cells (A375 vemR). However, no significant reduction in tumour size was observed (data not shown). It is speculated that compounds need to be modified to be more active, or that cancer‐specific compound delivery systems are needed.

Further studies are required to elucidate the detailed molecular mechanisms of these compounds and to optimize them as new therapeutic agents.

## 5 CONCLUSIONS

Our results showed that LL‐Z1640‐2 and patulin are promising compounds that decrease ZIC5 expression levels and induce apoptosis in various cancer cells, including melanoma, BRAF‐inhibitor resistant melanoma, pancreatic cancer and cholangiocarcinoma cells. Although the mechanism by which these compounds decrease ZIC5 expression levels remains unknown, our study indicates that LL‐Z1640‐2 and patulin have the potential to overcome unmet clinical needs.

## AUTHOR CONTRIBUTIONS

**Reiko Satow:** Conceptualization (lead); data curation (lead); formal analysis (lead); funding acquisition (lead); investigation (lead); methodology (lead); project administration (lead); supervision (lead); validation (lead); visualization (lead); writing – original draft (lead); writing – review and editing (lead). **Takeru Watanabe:** Data curation (equal); investigation (equal); methodology (equal). **Moeka Nomura:** Data curation (equal); investigation (equal); methodology (equal). **Shota Inagaki:** Data curation (supporting); investigation (supporting); methodology (supporting). **Atsuko Yoneda:** Data curation (supporting); investigation (supporting); writing – review and editing (supporting). **Kiyoko Fukami:** Conceptualization (supporting); funding acquisition (supporting); investigation (supporting); writing – review and editing (supporting).

## CONFLICT OF INTEREST

The authors have no conflicts of interest to declare.

## Data Availability

The data sets used during the current study are available from the corresponding author on reasonable request.
